# Merkel Cell Carcinoma of Left Groin: A Case Report and Literature Review

**DOI:** 10.1155/2013/431743

**Published:** 2013-05-21

**Authors:** Ahmed Abu-Zaid, Ayman Azzam, Ahmed Al-Wusaibie, Maraei Bin Makhashen, Abdulaziz Jarman, Tarek Amin

**Affiliations:** ^1^College of Medicine, Alfaisal University, P.O. Box 50927, Riyadh 11533, Saudi Arabia; ^2^Department of General Surgery, Faculty of Medicine, Alexandria University, Alexandria 21526, Egypt; ^3^Department of Surgical Oncology, King Faisal Specialist Hospital and Research Center (KFSH&RC), P.O. Box 3354, Riyadh 11211, Saudi Arabia; ^4^Department of Pathology and Laboratory Medicine, King Faisal Specialist Hospital and Research Center (KFSH&RC), P.O. Box 3354, Riyadh 11211, Saudi Arabia; ^5^Department of Plastic Surgery, King Faisal Specialist Hospital and Research Center (KFSH&RC), P.O. Box 3354, Riyadh 11211, Saudi Arabia

## Abstract

Merkel cell carcinoma (MCC) is an uncommon highly aggressive skin malignancy with an increased tendency to recur locally, invade regional lymph nodes, and metastasize distally to lung, liver, brain, bone, and skin. The sun-exposed skin of head and neck is the most frequent site of involvement (55%). We report the case of a 63-year-old Caucasian male patient who presented with a recurrent left inguinal mass for the third time after surgical resection with safe margins and no postoperative radio- or chemotherapy. The presented mass was excised, and pathological diagnosis revealed recurrent MCC. The patient underwent postoperative radiation therapy, and 6 months later, he developed a right groin mass which was resected and pathological diagnosis confirmed metastatic MCC. Six months later, patient developed an oropharyngeal mass which was unresectable, and pathological biopsy confirmed metastatic MCC. Patient was offered palliative radio- and chemotherapy. In this paper, we also present a brief literature review on MCC.

## 1. Introduction

Merkel cell carcinoma (MCC) is an uncommon highly aggressive skin malignancy that originates from the neuroendocrine and mechanoreceptor Merkel cells in the skin [1]. Clinical features of MCC are summed up by the acronym AEIOU: asymptomatic/nontender tumor, expanding rapidly, immune system suppression, older than 50 years, and ultraviolet-exposed/fair-skinned location [2]. The sun-exposed skin of head and neck is the most frequent location of involvement (55%) [3]. Due to its rarity and early asymptomatic clinical course, diagnosis of MCC is fairly challenging, often delayed, or even missed [4]. Definitive diagnosis requires a high index of clinical suspicion and most importantly skin biopsy for pathological examination. Majority of MCC patients present with localized disease (70–80%). The clinical course of MCC is highly aggressive with an increased predisposition to recur locally (26–60%), invade regional lymph nodes (45–91%), and metastasize distally (18–52%) [4] to lung, liver, brain, bone, and skin [5]. Management and prognosis of MCC are largely dependent on tumor staging at the time of presentation. Management modalities include utilization of surgical excision with safe margins, lymphadenectomy, radiotherapy, and chemotherapy [4]. Generally, prognosis of MCC is extremely poor with a high mortality rate [3]. 

Herein, we report a 63-year-old Caucasian male patient who presented with an unusual recurrent mass in the left groin (nonsun-exposed site) for the third time after surgical resection and subsequently developed regional metastasis to the contralateral groin, as well as distant metastasis to the oropharynx—an exceedingly unusual site of metastasis.

## 2. Case Report

A 63-year-old Caucasian male patient was referred to our hospital for further management of a recurrent big mass in the left inguinal region. Past medical history was remarkable for severely uncontrolled diabetes mellitus and hypertension. Past surgical history was remarkable for two surgical resections (with safe margins) of recurrent left inguinal masses and without postoperative radio- or chemotherapy. Pathological diagnosis of both resected masses revealed Merkel cell carcinoma. On physical examination, the left inguinal mass was oval, measuring around 9 × 11 cm, lobulated, nontender, firm, fixed to underlying tissue, and with no overlying skin changes. The patient was admitted for further tumor workup. 

Upon admission, all laboratory tests including complete blood count, renal, bone, hepatic, and coagulation profiles, carcinoembryonic antigen (CEA), alfa-fetoprotein (AFP), and CA 12–5 were normal. 

**Figure 1:**
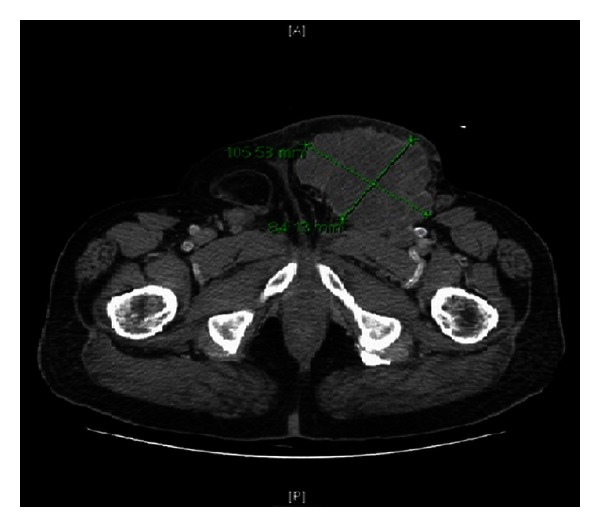
Computed tomography (CT) scan with contrast showing an 8.5 × 10.5 cm, heterogeneous, lobulated, and large mass in the left groin, compressing the left common femoral vein and inseparable from the vein as well as from the adductor muscles ventrally. The mass is associated with local lymphadenopathy, multiple small subcutaneous nodules, and an enlarged left external iliac lymph node.

Computed tomography (CT) scan with contrast revealed a large multilobulated mass with heterogeneous enhancement at the left groin. The mass was compressing the left common femoral vein and remained inseparable from the vein as well as from the adductor muscles ventrally. The mass was associated with local lymphadenopathy, multiple small subcutaneous nodules, and an enlarged left external iliac lymph node measuring around 1 × 1.5 cm (Figure 1).

**Figure 2:**
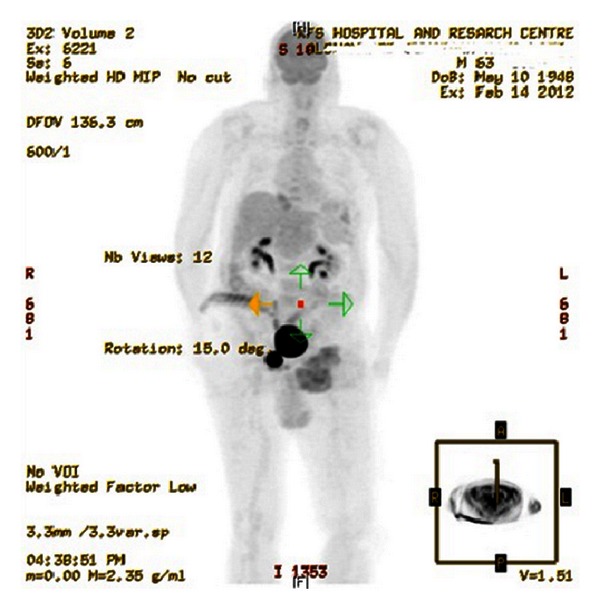
Positron emission tomography (PET) scan showing left inguinal hypermetabolic, heterogeneous, and lobulated mass lesion with few nodal lesions in the same vicinity consistent with the known Merkel cell carcinoma.

Positron emission tomography (PET) scan revealed hypermetabolic, heterogeneous, and lobulated lesion seen in the left groin that measured approximately 9.6 × 9 cm in its transverse and anteroposterior diameters. In the vicinity, there were few nodal lesions with moderate activity, mostly related to local metastatic disease (Figure 2).

**Figure 3:**
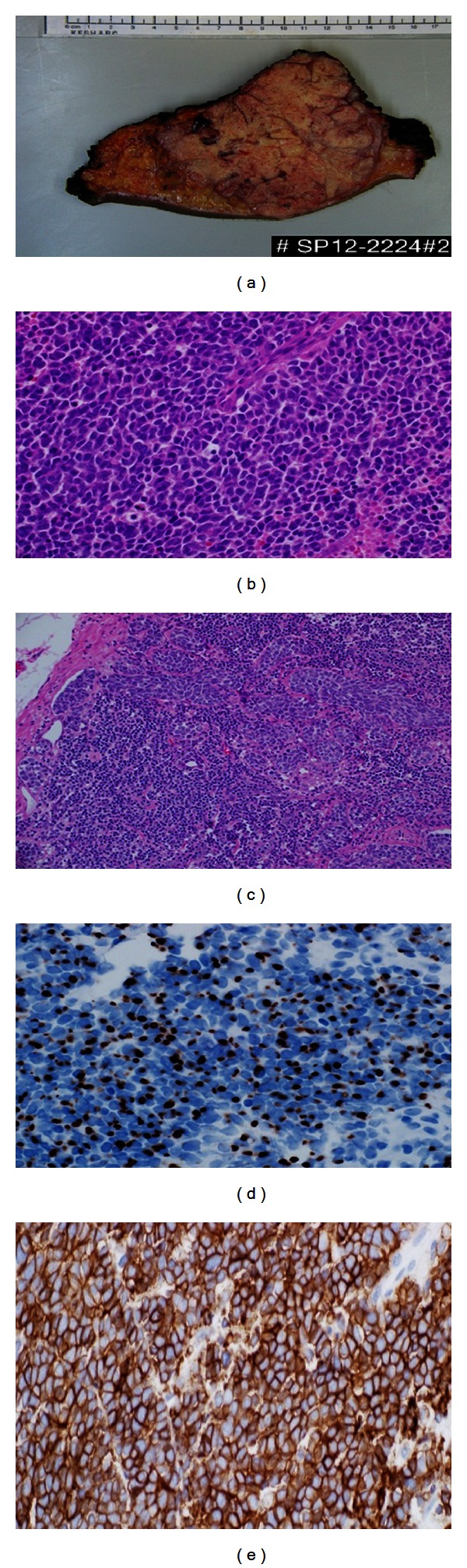
Merkel cell carcinoma. (a) Macroscopic examination of the resected mass showing a large, yellow-tanned, and lobulated mass. (b) H&E stain showing sheets of small blue cells with scant cytoplasm, irregular nuclei, mitoses, and individually necrotic cells. (c) H&E stain showing invasion of femoral lymph node by nests of neoplastic cells. (d) The neoplastic cells stain positive for CK20 in a perinuclear dot-like fashion. (e) The neoplastic cells stain positive for CD 56.

Afterwards, the patient underwent left inguinal dissection with excision of the tumor. Macroscopic examination revealed a large, solid, firm, yellow-tanned, and lobulated mass measuring 11 × 10.5 × 9.5 cm (Figure 3(a)). Microscopic examination showed a tumor composed of small uniformly sized blue neoplastic cells with round to oval nuclei, scant cytoplasm, distinct nuclear membranes, finely dispersed nuclear chromatin, and inconspicuous nucleoli (Figure 3(b)). Mitotic figures and individually necrotic cells were present. In addition, nests of neoplastic cells metastasizing to the left femoral lymph node were noted (Figure 3(c)). The neoplastic cells expressed cytokeratin 20 (CK20) in a perinuclear dot-like fashion (Figure 3(d)). Further, the neoplastic cells also expressed CD56 showing cytoplasmic and membranous positivity (Figure 3(e)). The neoplastic cell stained negative for LCA, S-100, CK7, and TTF-1. Based on the immunohistochemical stains, diagnosis of Merkel cell carcinoma (MCC) was established. Subsequently, the radiation oncology team was consulted, and the plan was to start radiotherapy 3 weeks after the operation.*‬‬*

Six months after hospital discharge and during the followup period, a rapidly growing mass appeared on the right groin, and the patient was admitted to the hospital. Local resection of the mass was done with safe margins. The postoperative period was uneventful. Pathology analysis revealed metastatic Merkel cell carcinoma. The patient was discharged in good shape and started on radiotherapy 1 month after hospital discharge.

Another 6 months after hospital discharge, the patient presented to the emergency department complaining of dysphagia with solid food and associated with muffled sound and throat pain. Consultation with ear, nose, and throat (ENT) team was done, and CT scan was ordered which revealed large exophytic mass lesion in the oropharynx arising from the left side base of the tongue indicative of malignant tumor with enlarged left-sided group 2 and 3 cervical lymphadenopathy with necrosis. PET scan revealed interval development of multiple fluorodeoxyglucose- (FDG-) avid lesions involving left thigh, left inguinal region, left hip, and left lower abdomen, consistent with progression of the MCC disease. Furthermore, activity was also noted in the lungs and neck bilaterally, suggestive of MCC metastases. Under general anesthesia, biopsy was taken from the large exophytic mass lesion in the oropharynx which proved to be metastatic Merkel cell carcinoma, and it was not amenable for resection. Tracheostomy and feeding gastrostomy tubes were inserted. The decision of the medical oncology consultation team was to start the patient on palliative radio- and chemotherapy. 

## 3. Discussion

Merkel cell carcinoma (MCC) is also known as primary small cell carcinoma of skin, primary neuroendocrine carcinoma of skin, primary undifferentiated carcinoma of skin, anaplastic carcinoma of skin, trabecular carcinoma of skin, and cutaneous APUDoma [6]. MCC is an exceedingly rare and highly aggressive cutaneous malignancy arising from uncontrolled proliferation of the neuroendocrine and mechanoreceptor Merkel cells that are located in the stratum basale layer of epidermal skin [1].

Numerous etiological factors contribute to development of MCC. These factors include exposure to ultraviolet (UV) radiation [7], infection with Merkel cell polyomavirus (MCV) [8], and statues of chronic immunosuppression [2, 9, 10]. MCC is mainly a malignancy of UV-exposed and fair-skinned elderly Caucasians [2]. It is most frequently found on skin-damaged and sun-exposed areas particularly face, head, and neck (55%), followed by extremities (40%) and lastly followed by truncal structures (5%) [3]. MCC occurring in nonsun-exposed sites, such as groins, is extremely uncommon. Although infection with MCV and its subsequent monoclonal integration into the genome accounts for approximately 80% of all cases of MCC [11], interestingly, our patient tested negative for anti-MCV antibodies. Immunosuppressed individuals who are at an increased risk of developing MCC include solid organ transplant recipients [9], HIV-infected AIDS patients [10], and B-cell lymphoma individuals [2].

Clinically, MCC presents as a painless, nontender, firm, glossy, bluish-red or bluish-purple, rapidly growing nodule of less than 2 cm in diameter at the time of clinical presentation [12]. Overlying skin may exhibit acneiform, telangiectatic, or ulcerative characteristics. In addition, associations with several satellite lymphadenopathies secondary to MCC invading dermal lymphatics are also possible [13]. Majority of MCC cases present as localized disease (70%–80%), followed by invasion of regional lymph nodes (9%–26%) and lastly followed by extra nodal distant metastasis (1%–4%) [4].

Due to the low incidence rate of MCC and its distinctive early symptom-free clinical course, diagnosis is highly challenging and therefore often delayed, or even missed [4]. Diagnosis is based on a hybrid of light microscopy, electron microscopy, and immunohistochemistry. Microscopically, MCC frequently originates in the dermis and occasionally extends into subcutaneous tissues and muscles; the overlying epidermis is usually preserved [14]. Histologically, the carcinoma is composed of small round blue cells, with sparse cytoplasm, medium- to large-sized hyperchromatic nuclei, multiple small nucleoli, delicately granular chromatin, abundant mitoses, and numerous apoptotic figures [4]. Ultrastructurally, electron microscopy demonstrates distinctive intracytoplasmic neurosecretory/neuroendocrine granules [1, 2] and collection of intermediate filaments organized in a globular paranuclear configuration [15].

MCC is occasionally mistaken for other histologically related cutaneous tumors, such as malignant melanoma, lymphoma, small cell lung carcinoma, and extraskeletal primitive neuroendocrine tumors (PNET/Ewing's sarcoma) [13]. Immunohistochemistry is a valuable method to establish a definitive differentiation between MCC and other closely related skin neoplasms [16]. Generally, MCC cells express both epithelial and neuroendocrine markers. Specifically, MCC cells stain positively for epithelial markers such as CK20 in a peculiar “dot-like” fashion (negative in extraskeletal PNET/Ewing's sarcoma) and stain negatively for epithelial markers such as LCA (positive in malignant lymphoma), S-100 (positive in malignant melanoma), and CK7 and TTF-1 (positive in small cell lung carcinoma) [17]. Furthermore, MCC cells stain positively for neuroendocrine markers such as chromogranin A, synaptophysin, NSE, neurofilament, and CD 56.

MCC is a highly aggressive cutaneous tumor with an increased tendency to recur locally (27%–60%), invade regional lymph nodes (45%–91%), and metastasize distally (18%–52%) [4]. MCC distant metastases are not uncommon and typically involve lung, liver, bone, brain, and skin [5]. MCC distant metastases to oropharyngeal structures are exceedingly rare.

There is no universally agreed consensus on staging of MCC [4]. However, the most broadly used staging system has been proposed by Yiengpruksawan et al. [18] based on clinical manifestations at the time of diagnosis, as follows: Stage I: localized skin tumor with no evidence of regional lymph nodes (IA: less than 2 cm, and IB: more than 2 cm); Stage II: evidence of regional lymph node invasion; Stage III: evidence of distant metastatic disease. Management of MCC primarily depends on the staging of disease at time of diagnosis: for Stages I and II, the aim of management is therapeutic, whereas for Stage III, the aim of management is geared towards palliative care.

For a localized disease (Stage I), an extensive surgical excision (3 cm wide and 2 cm deep), or alternatively, Mohs micrographic surgery [4, 19], along with adjuvant locoregional radiation therapy has been shown to yield an overall improved survival rather than surgery alone [20]. For patients with positive regional lymphadenopathy (Stage II), management is dependent on resectability of regional lymph nodes. Patients with possibly resectable lymph nodes are managed with regional lymph node dissection followed by adjuvant radiation therapy. On the contrary, patients with unresectable metastatic lymph nodes are treated with neoadjuvant radio- and/or chemotherapy followed by lymph node dissection [4]. Lymphadenectomy is highly recommended in sentinel lymph node- (SLN-) positive biopsies as SLN positivity is highly extrapolative of an increased risk of potential local/regional recurrence and distant metastasis [21]. On the contrary, prophylactic lymphadenectomy is neither recommended as a standard management scheme nor in SLN-negative biopsies; however, it is recommended in MCC malignancies that are at an increased risk of potential recurrence and aggressive metastasis [22].

For an MCC metastatic disease (Stage III), radio- and chemotherapy are employed with a palliative intent [23]. MCC is predominantly a radio-sensitive malignancy, and utilization of radiation therapy is highly advised [24]. However, the role of chemotherapy is still debatable and does not seem to yield survival benefit [3]. Tumor regression and remission rates can be as high as 70%; however, disease rapidly recurs within a couple of months, and response does not appear to significantly prolong survival [3]. The most frequently used chemotherapeutic regimens include the combination of cyclophosphamide, doxorubicin, and vincristine, as well as the combination of cisplatin or carboplatin plus/minus etoposide [3, 25].

Generally, prognosis of MCC is unfortunate with a high mortality rate in which nearly one-third of patients pass away from MCC within 36 months from the time of diagnosis [3]. The following clinical and histological factors are predictive of poor prognosis in MCC: male gender, age more than 65 years, existence of comorbid immunocompromised/immunosuppressant status, presence of metastatic disease, tumor situated in head and neck regions, tumor size more than 2 cm in diameter, tumor with more than 10 mitoses per high-power field (HPF), evidence of vascular invasion, absence of an inflammatory reaction, and high expression of Ki-67 (proliferation index marker) and p63 (antiproliferative and apoptosis-inducing marker) [1, 26].

The most significant unfavorable/poor prognostic factor of long-term survival and also associated with an increased risk of yielding metastatic disease is an evidence of lymph node invasion [19]. Presence of regional lymph node invasion markedly drops the overall survival rate from 90% to 50%, and it occurs in almost 50%–70% of all patients within 24 months from the time of clinical diagnosis [21]. Presence of distant metastases signifies a deadly condition, and the anticipated survival is frequently less than 10% within a frame time of 3 years [27], and death mostly ensues within 10 months from time of diagnosis of metastatic disease [28]. The literature has shown that neither chemotherapeutic nor immunotherapeutic or molecular-targeted therapeutic tactics have revealed favorable impact on the management of metastatic MCC disease [29].

## 4. Conclusion

Merkel cell carcinoma (MCC) occurring in nonsun-exposed sites, such as inguinal regions, is exceptionally rare. However, they should always be considered in the differential diagnosis in any elderly (above 50 years old), fair-thinned and immunocompromised patient presenting with a nontender and rapidly expanding inguinal mass. Skin biopsy for pathological examination is necessary for a definitive diagnosis. Management of MCC is largely dependent on tumor staging at the time of presentation: curative intent for locoregional disease and palliative intent for distant disease. Optimal management modalities with varying results include surgical excision of primary tumor with safe margins, radiotherapy, chemotherapy, immunotherapy, molecular-targeted therapy, and lymphadenectomy to control regional and distant disease. Despite management, MCC has an exceptionally increased tendency to recur locally (26–60%), invade regional lymph nodes (45–91%), and eventually metastasize distally (18–52%) to liver, lung, bone, brain, and skin. Hence, frequent short- and long-term followups are highly recommended. Broadly, MCC has a very poor prognosis, and generally one-third of patients pass away from MCC within 36 months from time of diagnosis.

## References

[1] Gollard R, Weber R, Kosty MP, Greenway HT, Massullo V, Humberson C (2000). Merkel cell carcinoma review of 22 cases with surgical, pathologic, and therapeutic considerations. *Cancer*.

[2] Heath M, Jaimes N, Lemos B (2008). Clinical characteristics of Merkel cell carcinoma at diagnosis in 195 patients: the AEIOU features. *Journal of the American Academy of Dermatology*.

[3] Rockville Merkel cell Carcinoma Group (2009). Merkel cell carcinoma: recent progress and current priorities on etiology, pathogenesis, and clinical management. *Journal of Clinical Oncology*.

[4] Pectasides D, Pectasides M, Economopoulos T (2006). Merkel cell cancer of the skin. *Annals of Oncology*.

[5] Peloschek P, Novotny C, Mueller-Mang C (2010). Diagnostic imaging in Merkel cell carcinoma: lessons to learn from 16 cases with correlation of sonography, CT, MRI and PET. *European Journal of Radiology*.

[6] Haag ML, Glass LF, Fenske NA (1995). Merkel cell carcinoma: diagnosis and treatment. *Dermatologic Surgery*.

[7] Fernández-Figueras MT, Puig L, Musulén E (2007). Expression profiles associated with aggressive behavior in Merkelcell carcinoma. *Modern Pathology*.

[8] Feng H, Shuda M, Chang Y, Moore PS (2008). Clonal integration of a polyomavirus in human Merkel cell carcinoma. *Science*.

[9] Koljonen V, Kukko H, Tukiainen E (2009). Incidence of Merkel cell carcinoma in renal transplant recipients. *Nephrology Dialysis Transplantation*.

[10] Engels EA, Frisch M, Goedert JJ, Biggar RJ, Miller RW (2002). Merkel cell carcinoma and HIV infection. *The Lancet*.

[11] Houben R, Shuda M, Weinkam R (2010). Merkel cell polyomavirus-infected Merkel cell carcinoma cells require expression of viral T antigens. *Journal of Virology*.

[12] Swann MH, Yoon J (2007). Merkel cell Carcinoma. *Seminars in Oncology*.

[13] Gorjón PS, Martín ACM, Pérez PB, González JLG, del Pozo de Dios JC, Melgar A (2011). Merkel cell carcinoma: a presentation of 5 cases and a review of the literature. *Acta Otorrinolaringológica Española*.

[14] Bayrou O, Avril MF, Charpentier P, Caillou B, Guillaume JC, Prade M (1991). Primary neuroendocrine carcinoma of the skin. Clinicopathologic study of 18 cases. *Journal of the American Academy of Dermatology*.

[15] Hierro I, Blanes A, Matilla A, Muñoz S, Vicioso L, Nogales FF (2000). Merkel cell (neuroendocrine) carcinoma of the vulva. A case report with immunohistochemical and ultrastructural findings and review of the literature. *Pathology Research and Practice*.

[16] Gessner K, Wichmann G, Boehm A (2011). Therapeutic options for treatment of Merkel cell carcinoma. *European Archives of Oto-Rhino-Laryngology*.

[17] Nghiem P, Jaimes N, Wolff K, Katz S, Goldsmith L, Gilchrest B, Leffell D, Paller A (2008). Chapter 120: Merkel cell carcinoma. *Fitzpatrick’s Dermatology in General Medicine*.

[18] Yiengpruksawan A, Coit DG, Thaler HT, Urmacher C, Knapper WK (1991). Merkel cell carcinoma: prognosis and management. *Archives of Surgery*.

[19] Eng TY, Boersma MGK, Fuller CD, Cavanaugh SX, Valenzuela F, Herman TS (2004). Treatment of Merkel cell carcinoma. *American Journal of Clinical Oncology*.

[20] Veness MJ (2006). Merkel cell carcinoma (primary cutaneous neuroendocrine carcinoma): an overview on management. *Australasian Journal of Dermatology*.

[21] Mehrany K, Otley CC, Weenig RH, Phillips PK, Roenigk RK, Nguyen TH (2002). A meta-analysis of the prognostic significance of sentinel lymph node status in Merkel cell carcinoma. *Dermatologic Surgery*.

[22] Ratner D, Nelson BR, Brown MD, Johnson TM (1993). Merkel cell carcinoma. *Journal of the American Academy of Dermatology*.

[23] Papamichail M, Nikolaidis I, Nikolaidis N (2008). Merkel cell carcinoma of the upper extremity: case report and an update. *World Journal of Surgical Oncology*.

[24] Meeuwissen JA, Bourne RG, Kearsley JH (1995). The importance of postoperative radiation therapy in the treatment of Merkel cell carcinoma. *International Journal of Radiation Oncology Biology Physics*.

[25] Viola G, Visca P, Bucher S, Migliano E, Lopez M (2006). Merkel cell carcinoma. *Clinica Terapeutica*.

[26] Wong HH, Wang J (2010). Merkel cell carcinoma. *Archives of Pathology & Laboratory Medicine*.

[27] Allen PJ, Bowne WB, Jaques DP, Brennan MF, Busam K, Coit DG (2005). Merkel cell carcinoma: prognosis and treatment of patients from a single institution. *Journal of Clinical Oncology*.

[28] Smith DF, Messina JL, Perrott R (2000). Clinical approach to neuroendocrine carcinoma of the skin (Merkel cell carcinoma). *Cancer Control*.

[29] Desch L, Kunstfeld R (2013). Merkel cell carcinoma: chemotherapy and emerging new therapeutic options. *Journal of Skin Cancer*.

